# High-level production of the agmatine in engineered *Corynebacterium crenatum* with the inhibition-releasing arginine decarboxylase

**DOI:** 10.1186/s12934-022-01742-3

**Published:** 2022-01-31

**Authors:** Fengyu Yang, Jiayu Xu, Yichun Zhu, Yi Wang, Meijuan Xu, Zhiming Rao

**Affiliations:** Key Laboratory of Industrial Biotechnology of the Ministry of Education, School of Biotechnology, Jiangnan University, Wuxi, 214122 Jiangsu China

**Keywords:** *Corynebacterium crenatum*, Agmatine, Arginine decarboxylase, Rational design, Feedback inhibition

## Abstract

**Background:**

Agmatine is a member of biogenic amines and is an important medicine which is widely used to regulate body balance and neuroprotective effects. At present, the industrial production of agmatine mainly depends on the chemical method, but it is often accompanied by problems including cumbersome processes, harsh reaction conditions, toxic substances production and heavy environmental pollution. Therefore, to tackle the above issues, arginine decarboxylase was overexpressed heterologously and rationally designed in *Corynebacterium crenatum* to produce agmatine from glucose by one-step fermentation.

**Results:**

In this study, we report the development in the Generally Regarded as Safe (GRAS) l-arginine-overproducing *C. crenatum* for high-titer agmatine biosynthesis through overexpressing arginine decarboxylase based on metabolic engineering. Then, arginine decarboxylase was mutated to release feedback inhibition and improve catalytic activity. Subsequently, the specific enzyme activity and half-inhibitory concentration of I534D mutant were increased 35.7% and 48.1%, respectively. The agmatine production of the whole-cell bioconversion with AGM3 was increased by 19.3% than the AGM2. Finally, 45.26 g/L agmatine with the yield of 0.31 g/g glucose was achieved by one-step fermentation of the engineered *C. crenatum* with overexpression of *speA*_I534D_.

**Conclusions:**

The engineered *C. crenatum* strain AGM3 in this work was proved as an efficient microbial cell factory for the industrial fermentative production of agmatine. Based on the insights from this work, further producing other valuable biochemicals derived from l-arginine by *Corynebacterium crenatum* is feasible.

**Supplementary Information:**

The online version contains supplementary material available at 10.1186/s12934-022-01742-3.

## Background

Agmatine, also known as (4-aminobutyl) guanidine, one of the important biogenic amines, is widely applied in a variety of industrial fields such as pharmaceuticals [1], food, chemicals and feed [2]. As an important essence of medicine or nutrition, agmatine can enhance metabolism, regulate nutritional balance or accelerate body recovery and has gained interest from the medical industry [3, 4]. In addition, agmatine has been shown to have neuroprotective effects in cases such as stroke, central nervous system traumas and neuropathic pain [5, 6]. At present, the industrial production of agmatine mainly depends on the chemical method, which is often accompanied by problems such as low efficiency, harsh reaction conditions and heavy environmental pollution [7]. With the growing world market for agmatine, there is a significantly increased interest in the more efficient and economical production of agmatine. Therefore, it is necessary to find a safe and environmental-friendly biological approach for the production of agmatine.

The reaction from l-arginine to agmatine is catalyzed with the enzyme arginine decarboxylase (ADC; EC 4.1.1.19) encoded by the *speA* or *adiA* gene [8, 9]. Currently, *Escherichia coli* is an attractive microbial chassis to produce various substances by metabolic engineering, since it has a clear genetic background [10–12]. Xu et al. had constructed a series of engineered *E. coli* for agmatine production [13, 14]. They first constructed an engineered *E. coli* for agmatine production through the deletion of *speC* and *speF* (encoding the ornithine decarboxylase isoenzymes), *speB* (encoding agmatinase) and *argR* (encoding a transcriptional repressor). And then, the arginine decarboxylase gene *speA* from *E. coli* was overexpressed in the base strain AUX4, generating the strain AUX5. The engineered strain *E. coli* AUX5 produced 15.32 g/L agmatine with a yield of 0.11 g/g glucose in fed-batch fermentation [13]. Xu et al. used heterologous strong promoters sequentially to overexpress the genes encoding glutamate dehydrogenase (*gdhA*), glutamine synthetase (*glnA*), phosphoenolpyruvate carboxylase (*ppc*), aspartate aminotransferase (*aspC*), transhydrogenase (*pntAB*), and l-arginine decarboxylase (*speA*) in the previously developed *E. coli* strain AUX4. They finally constructed an engineered *E. coli* strain AUX11 by metabolic engineering, which produced 40.43 g/L of agmatine with a yield on the glucose of 0.29 g/g [14]. In addition, the inducible arginine decarboxylase AdiA were overexpressed in *E. coli* at a 15-L fermenter and 279.21 g/L of agmatine was produced by whole-cell biotransformation with a conversion rate of 98% [15].

**Fig. 1:**
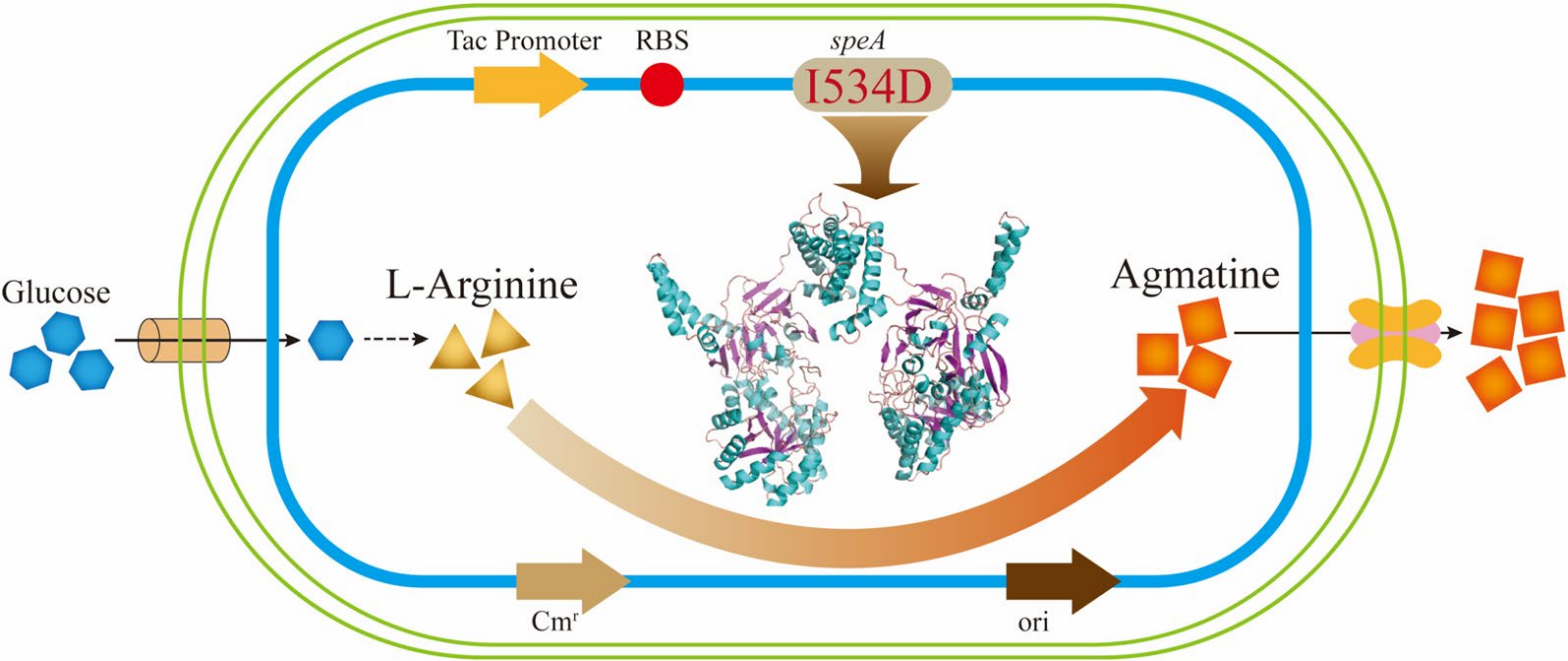
Biosynthetic pathway from l-arginine to agmatine

*Corynebacterium crenatum* is a Gram-positive bacterium and had been recognized as a well-established host to produce amino acids [16], chemicals [17], and natural products [18]. In contrast to *E. coli*, *C. crenatum*, a Generally Regarded as Safe (GRAS) strain, was the most commonly used industrial microorganism for the production of various amino acids and derivatives, including l-glutamate, l-arginine, and l-lysine [19]. Furthermore, *C. crenatum* possesses a strong metabolic flux to l-glutamate, making it a more potent producer of l-arginine and its derivatives [20]. In our previous study, the base *C. crenatum* SYPA was developed and shown to be capable to overproduce l-arginine [21, 22]. Based on the above description, we are committed to broadening the product spectrum of *Corynebacterium crenatum*.

In this study, we report a metabolically engineered *C. crenatum* strain for enhancement the production of agmatine by overexpressing the arginine decarboxylase (ADC) on the recombinant plasmid (Fig. 1). To release agmatine inhibition of SpeA and improve the titer and yield of agmatine, we generated 16 mutants by site-directed mutagenesis. The best result showed that the half-inhibitory concentration and specific enzyme activity of the I534D mutant were 48.1% and 35.7% higher than those of the wild-type SpeA. Meanwhile, the agmatine production of whole-cell bioconversion with AGM3 was increased by 19.3% compared with AGM2. Finally, our engineered strain *C. crenatum* AGM3 was performed for synthesizing agmatine by fed-batch fermentation and allowed the production of 45.26 g/L with the yield of 0.31 g/g glucose. To the best of our knowledge, this is the first report for the production of agmatine by engineering *Corynebacterium crenatum*.

## Results

### Construction of the engineered *C. crenatum* by overexpressing arginine decarboxylase

Agmatine, an intermediate metabolite of the biogenic amines synthesis pathway, is produced from l-arginine as the precursor [23]. In our previous work, l-arginine concentration in the fermentation supernatant was increased via the inactivation of arginine decarboxylase, which could direct the precursor molecules for the production of agmatine [24]. The arginine decarboxylase from *E. coli* was selected to produce agmatine [25], and *C. crenatum* SYPA was chosen as the chassis strain in this work due to its capability to overproduce l-arginine. Hence, genes encoding arginine decarboxylase from *E. coli* were overexpressed heterologously in the host strain. Two ADCs exist in *E. coli*, encoded by the constitutively expressed *speA* and the inducible *adiA*. AdiA is an inducible enzyme which is induced in a low pH growth environment [26], while SpeA is a constitutive enzyme and plays a role in the biosynthesis of agmatine [27]. The gene with a higher level of specific arginine decarboxylase activity among *speA* and *adiA* will be selected for the production of agmatine.

To effectively produce agmatine by *C. crenatum* SYPA, the shuttle plasmid pXMJ19 with an inducible strong *tac* promoter was selected as the vector. We cloned the ADC-coding gene into the pXMJ19 plasmid to construct a recombinant ADC expression plasmid. And then, the expression vectors were transformed into *C. crenatum* SYPA to generate two recombinant strains, termed AGM1 (*C. crenatum* SYPA/pXMJ19-*adiA*) and AGM2 (*C. crenatum* SYPA/pXMJ19-*speA*). The empty vector pXMJ19 was introduced into *C. crenatum* SYPA to generate the control strain AGM0.

### Activity assay and characterization of recombinant arginine decarboxylase

After construction of the recombinant strains, the effect of overexpressing ADC on the agmatine production was analyzed using recombinant strains by whole-cell biotransformation. l-Arginine was added to the reaction mixture and was directly converted into agmatine by the recombinant strains with ADC, which can clearly reflect the activity of arginine decarboxylase. Table 1 shows that the two arginine decarboxylases can direct the l-arginine for the production of agmatine and the AGM0 was used as a control strain. A total of 21.72 g/L of agmatine in a 57.45% conversion ratio was produced from l-arginine using AGM2, while a lower conversion ratio of 40.74% was obtained with AGM1. As we expected, without any agmatine was produced by the strain AGM0, and the crude enzyme activity analyses also demonstrated the above result (Table 2). Although the recombinant strain with heterologous expression of the *speA* gene from *E. coli* showed higher levels of agmatine production, in order to select the better enzyme for the next research, the enzymatic properties of the two ADCs were determined.

**Table 1:** The agmatine production of strains with different ADCs by whole-cell biotransformation

Strains	Agmatine (g/L)	Conversion rate (%)
*C. crenatum* AGM0	ND	ND
*C. crenatum* AGM1	15.34 ± 0.22	40.74 ± 0.58
*C. crenatum* AGM2	21.72 ± 0.50	57.45 ± 0.84

ND means no data detected

**Table 2:** The arginine decarboxylase activities of crude extracts from engineering *C. crenatum* and purified enzyme from *E. coli*

Strains	Total activity (U/mL)	Specific activity (U/mg)
*C. crenatum* AGM0	ND	ND
*C. crenatum* AGM1	144.8 ± 6.8	8.3 ± 0.5
*C. crenatum* AGM2	177.7 ± 6.1	8.9 ± 0.3
Purified AdiA	92.9 ± 3.4	46.4 ± 1.9
Purified SpeA	105.8 ± 2.3	55.7 ± 1.2

ND means no data detected

**Fig. 2:**
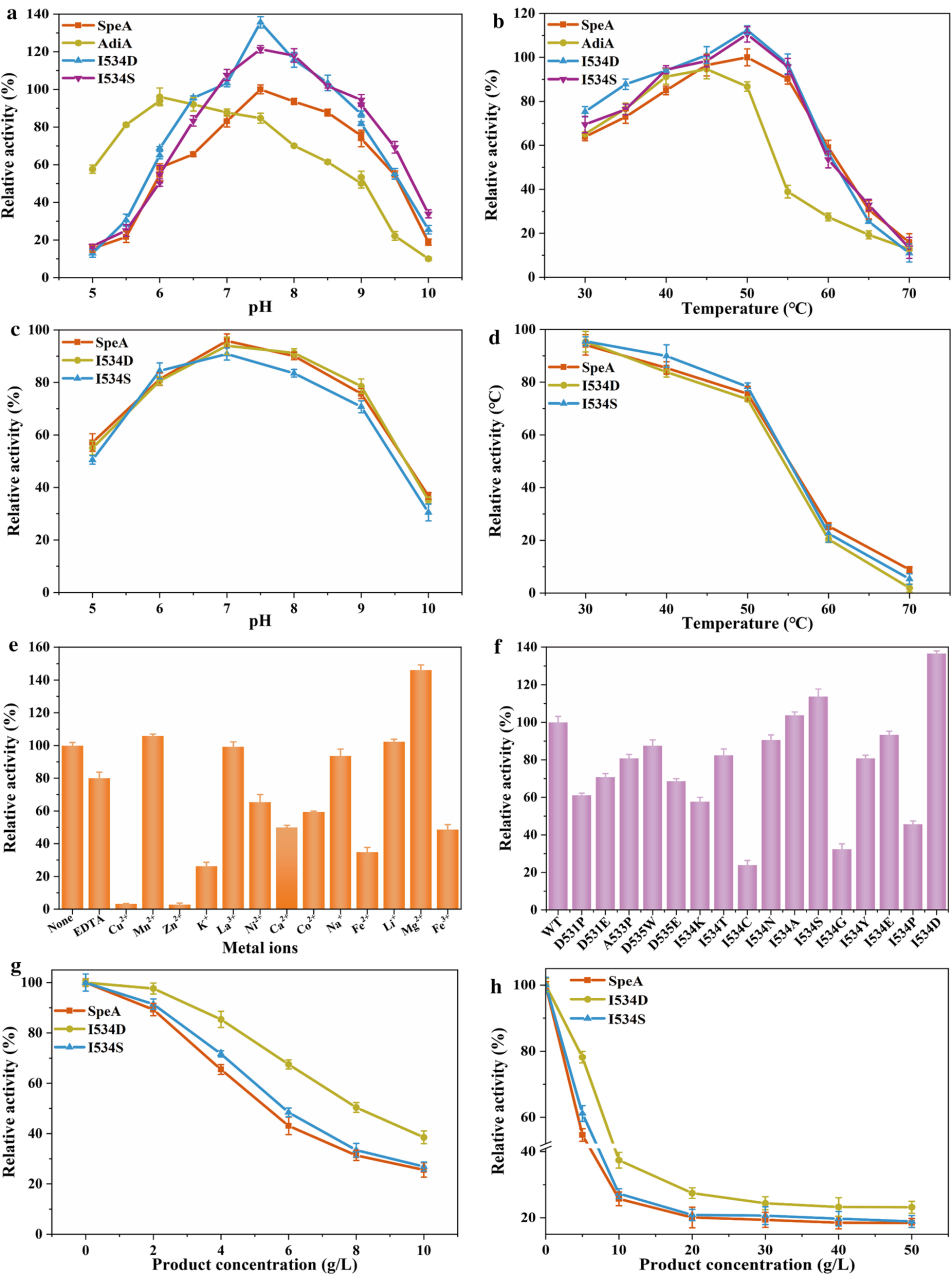
Biochemical characterization of ADCs and mutations. **a** Effect of pH on ADCs and mutations activity. **b** Effect of temperature on ADCs and mutations activity. **c** Residual activity of SpeA and its mutations after incubated for 12 h at different pH buffers. The initial activity before incubation was defined as 100%. **d** Residual activity of SpeA and its mutations after incubated for 12 h at different temperatures. The initial activity before incubation was defined as 100%. **e** Effect of metal ions on wild-type SpeA activity. **f** Specific enzyme activities of different SpeA variants. The wild-type SpeA enzyme activity was defined to 100%. **g** The enzyme activity of wild-type SpeA and mutations at different agmatine concentrations. **h** The enzyme activity of wild-type SpeA and mutations at high agmatine concentrations. The enzyme activity without agmatine was defined as 100%. All determinations were performed in triplicate

As shown in Table 2, arginine decarboxylase SpeA and AdiA were purified by His-Tag (His-Tag at C-terminal) and enzyme activity was determined. The specific activity of purified SpeA and AdiA were 55.7 U/mg and 46.4 U/mg, respectively. And the specific activity of purified SpeA was 6.2-fold higher than that of the crude cell extracts. Besides, pH and temperature are important parameters affecting enzyme activity and the result of whole-cell biotransformation and fermentation. Then, the properties of two arginine decarboxylases were further characterized by different temperatures and pH values. The optimum pH of ADC was measured from 5.0 to 10.0, and the maximum activity of SpeA was observed at pH 7.5 (Fig. 2a). In contrast, the maximum specific enzyme activity of AdiA appeared at pH 6.0. As described in Fig. 2b, the maximum activity of SpeA and AdiA was detected at 50, 45 °C, respectively, when we measured the optimum temperature from 30 to 70 °C. The catalytic activity of AdiA decreased significantly when the temperature exceeded 50 °C. Based on these findings, SpeA was used for agmatine production in proceeding studies. The effect of metal ions on SpeA activity was shown in Fig. 2e, the enzyme activity was promoted 46.09% by 1 mM Mg^2+^. At the same time, the feedback inhibition of the product was next determined. We determined the enzyme activity by adding different concentrations of product to the reaction system. As shown in Fig. 2g, the results showed that the half-inhibitory concentration of SpeA was 5.4 g/L. In order to enhance the catalytic efficiency, SpeA was rationally designed to release feedback inhibition.

### Target residue selection for mutation and function determination

**Fig. 3:**
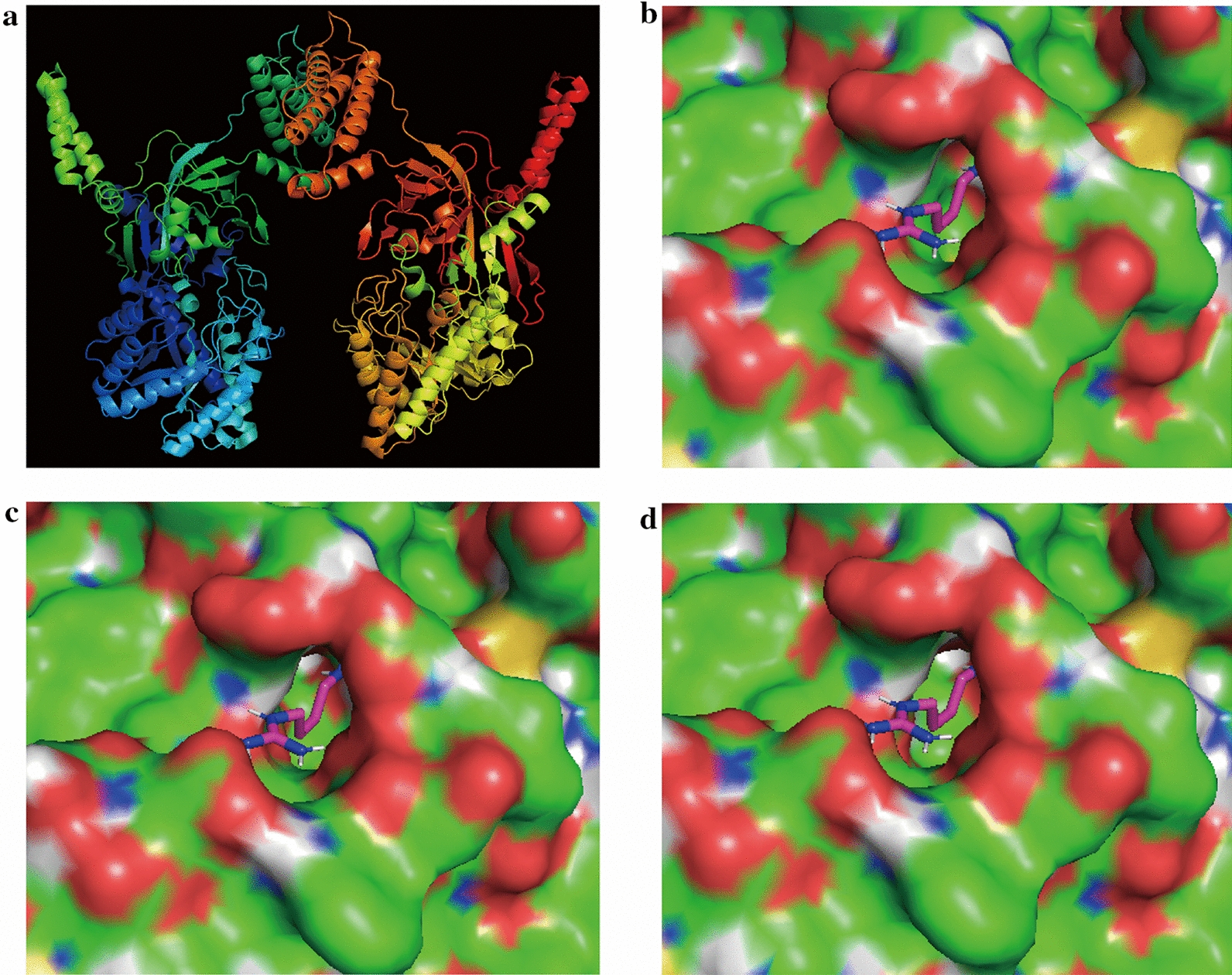
The structure of wild-type SpeA and the structural analysis of SpeA, I534D and I534S after docking agmatine. **a** The structure of wild-type SpeA. **b** Changes in product docking site on the surface of SpeA. **c** Changes in product docking site on the surface of I534D. **d** Changes in product docking site on the surface of I534S

The structure of arginine decarboxylase protein (PDB ID: 3NZQ) from *E. coli* was used as the initial enzyme structure. A tetramer was observed for arginine decarboxylase from *E. coli*, composed of two dimers of tightly associated monomers. The structure was visually displayed by Schrodinger, and the protein structure was pretreated (Fig. 3a). Then, the structure of arginine decarboxylase was docked with the molecule of agmatine that was pretreated. Mutations located in 531D, 533 A, 534I and 535D were close to the active center, may play a crucial role in releasing feedback inhibition. Virtual saturation mutagenesis library of four key amino acids was constructed by Schrodinger. The 16 mutants with improved prime stability were selected and listed in Additional file 1: Table S2. After the completion of site-directed mutagenesis, two mutants (I534D and I534S) with increased enzyme activity were identified by enzyme activity assay (Fig. 2f). The optimum temperature and pH of the two mutants were measured and found to be the same as that of the SpeA, except for an increase in enzyme activity. Catalytic activity decreased significantly when the temperature exceeded 60 °C (Fig. 2d). Meanwhile, two mutants and SpeA had more than 90% residual activity after incubation for 12 h under neutral conditions (Fig. 2c). As shown in Fig. 2g, the half-inhibitory concentration of I534D was increased from 5.4 g/L to 8.0 g/L compared with wild-type SpeA.

According to previous reports, residues which were adjacent to the active center played an important role in catalytic efficiency [28]. In our studies, the I534 site was near the active center, thus I534 acted a crucial role in catalytic activity. Thus, mutating the hydrophobic isoleucine (+4.5) into hydrophilic aspartic acid (– 3.5) decreased the affinity for the product agmatine, reducing feedback inhibition. Meanwhile, the mutation of serine (− 0.8), which was relatively less hydrophilic, also had a little effect on releasing feedback inhibition. In addition, the catalytic efficiency of mutant I534D was increased compared with wild-type SpeA, which may be attributed to the smaller side chain and the release of feedback inhibition after mutation [29] (Fig. 3c). Furthermore, the *Kcat*/Km of I534D was 1.42 times higher than SpeA, indicating that I534D significantly improved catalytic efficiency (Table 3). Although the substrate affinity of the I534D mutant was reduced, the *Kcat* was increased 1.48-fold seems to result from the enhanced product release. And our results can be supported by previous study [30].

**Table 3:** The kinetic parameters of wild-type SpeA and mutants towards substrates l-arginine

	WT	I534D	I534S
Km (mM)	0.602 ± 0.092	0.623 ± 0.075	0.691 ± 0.069
*Kcat* (S^−1^)	1.788 ± 0.135	2.643 ± 0.328	2.121 ± 0.206
*Kcat*/Km (S^−1^ mM^−1^)	2.97	4.242	3.069
IC_50_ (g/L)	5.4 ± 0.19	8.0 ± 0.21	5.9 ± 0.24
Ki (mM)	4.42 ± 0.32	6.59 ± 0.45	5.46 ± 0.39

The gene encoding mutant I534D was linked with the shuttle plasmid pXMJ19 to construct recombinant expression plasmid and it was transformed into strain *C. crenatum* SYPA to produce AGM3 (*C. crenatum* SYPA/pXMJ19-*speA*_I534D_).

### Recombinant whole-cell bioconversion for the production of agmatine

**Fig. 4:**
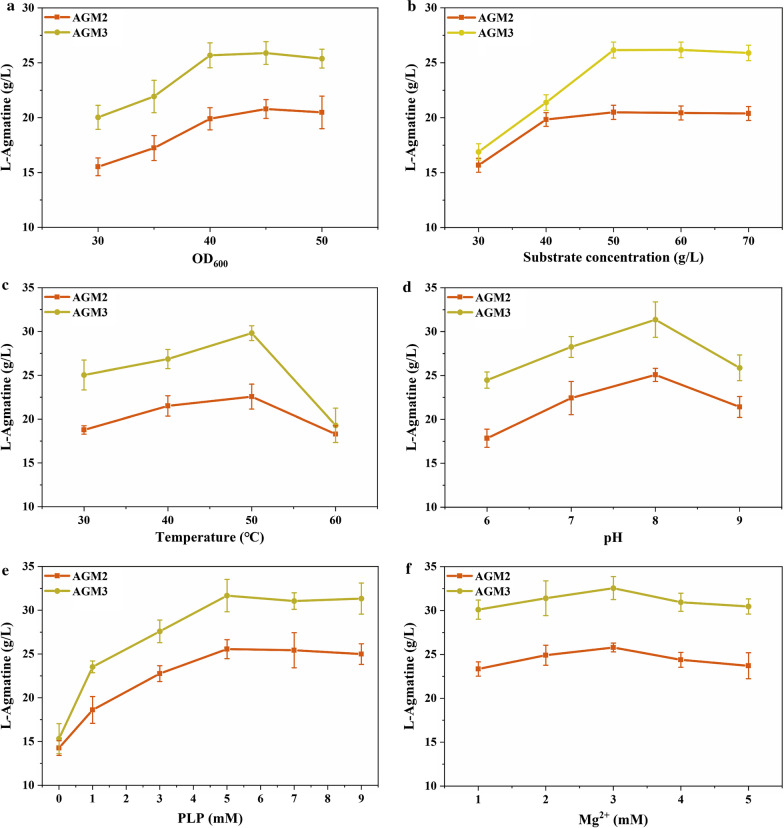
Optimization of whole-cell biotransformation conditions for agmatine production. **a** The cell density of the biotransformation system. **b** The substrate concentration of the biotransformation system. **c** The temperature of the biotransformation system. **d** The pH of the biotransformation system. **e** The PLP concentration of the biotransformation system. **f** The Mg^2+^ concentration of the biotransformation system. All determinations were performed in triplicate

A mutant that can reduce the inhibition of the product was selected through rational modification, and its catalytic activity was also increased compared with SpeA. The production of agmatine by whole-cell biotransformation was used to test the effect of l-arginine decarboxylase mutation [31]. The effect of the final OD_600_ value, substrate concentration, temperature, pH, PLP and Mg^2+^ on agmatine production was determined. The amount of cell in the transformation mixture is one of the crucial factors in the transformation reaction. As shown in Fig. 4a, when the final OD_600_ value of *C. crenatum* cells reached 40, the optimal agmatine titer was 25.68 g/L with a conversion rate of 68.19%. As shown in Fig. 4b, the optimal substrate concentration of AGM3 appears at 50 g/L, the conversion production decreased when other concentrations were added. When 40 g/L l-arginine was added, the agmatine yield of AGM2 was highest. By adjusting the temperature from 30 to 60 ℃, the maximum agmatine titer was observed at 50 ℃ for both AGM2 and AGM3 (Fig. 4c). The agmatine titer was increased gradually from 30 to 50 ℃ but decreased significantly from 50 to 60 ℃. The highest agmatine titer was achieved at 50 ℃, and the agmatine titer was 29.81 g/L at a conversion rate of 79.18%. We assayed the agmatine concentration using whole‑cell bioconversion by changing the pH from 6.0 to 9.0. At pH 8.0, the highest production of 31.37 g/L was formed and the titer of agmatine dropped with the pH decrease (Fig. 4d). At pH 9.0, the catalyst activity decreased and a lower titer of agmatine was formed. PLP is essential for arginine decarboxylase and the catalytic activity of arginine decarboxylase can be improved when PLP was added [32]. As shown in Fig. 4e, the catalysis efficiency was highest when 5 mM PLP was added to the transformation broth and 31.66 g/L agmatine could be produced in 12 h. The agmatine titer of the mutant increased from 30.11 to 32.55 g/L in 12 h from 50 g/L l-arginine at different Mg^2+^ concentrations (Fig. 4f). As the concentration of Mg^2+^ increased, the highest agmatine titer was achieved at 3 mM, and then agmatine decreased with increasing Mg^2+^ concentrations.

On the basis of the optimized transformation conditions described above, the reaction system was added 50 g/L l-arginine at 0 and 6 h. In short, the reaction mixture included 100 g/L l-arginine, 5 mM PLP, 3 mM Mg^2+^, the OD_600_ = 40 of induced cells and 50 mM Tris-HCl buffer (pH 8.0). Finally, the agmatine production of whole-cell bioconversion with AGM3 was 62.84 g/L at a conversion rate of 83.44% in 12 h, but only a lower level of 48.24 g/L agmatine was produced in recombinant strain AGM2 (Table 4).

**Table 4:** The whole-cell biotransformation on agmatine production under optimized conditions in *C. crenatum* AGM2 and AGM3

Strains	Agmatine (g/L)	Conversion rate (%)
*C. crenatum* AGM2	48.24 ± 1.61	64.05 ± 2.14
*C. crenatum* AGM3	62.84 ± 0.64	83.43 ± 0.85

### One-step preparation of agmatine from glucose was realized by *C. crenatum*

**Fig. 5:**
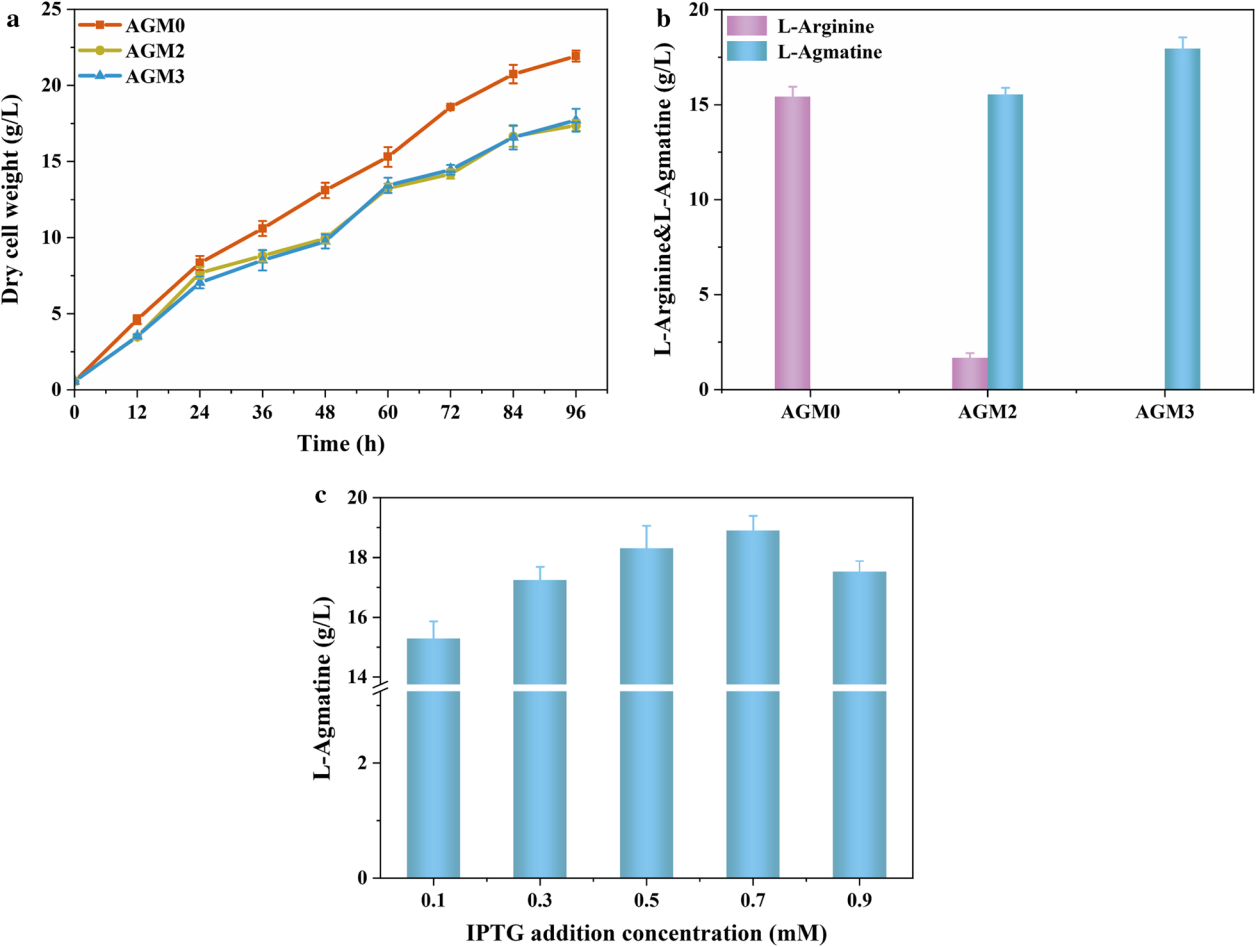
Effect of modifying arginine decarboxylase on agmatine production in shaking flask. **a** The dry cell weight of AGM0, AGM2 and AGM3. **b** Agmatine production of strains AGM0, AGM2 and AGM3. **c** Agmatine production of strain AGM3 by adding different concentrations of IPTG. All determinations were performed in triplicate

We engineered *C. crenatum* strain for the production of agmatine using l-arginine as the substrate that may compete with the use of l-arginine in pharmaceuticals and feed industries. Glucose, an inexpensive and attractive sustainable fermentation raw material, is an excellent raw material for the production of various high value-added compounds and was used for the production of agmatine to address this problem. AGM0 was used as the control strain, shaking flask cultures of AGM2 and AGM3 with glucose as substrate supply was carried out to compare the OD_600_ and agmatine titer of engineered strain *C. crenatum*. As shown in Fig. 5a, *C. crenatum* AGM0 grew with a rate of 0.23 h^−1^ to a final biomass concentration of 21.93 g/L. The overexpression of arginine decarboxylase affected the cell growth, resulting in the cell dry weight of AGM2 and AGM3 lower than that of the control strain. *C. crenatum* AGM3 grew with a rate of 0.18 h^−1^ to a final cell dry weight concentration of 17.71 g/L and the cell density measurement showed no significant differences between AGM2 and AGM3. These results indicate that AGM0 is unable to produce agmatine, although the cell density of AGM0 was higher than the other two strains (Fig. 5b). As described in Fig. 5b, a total of 15.53 g/L of agmatine was produced from glucose using *C. crenatum* AGM2, while a high level of agmatine was obtained with AGM3. Agmatine was produced to a titer of 17.96 g/L corresponding to a yield of 0.16 g/g glucose by *C. crenatum* AGM3. After fermentation, the strain AGM2 produced 1.68 g/L l-arginine, which was not found in AGM3, which may be due to the relatively low catalytic activity and the feedback inhibition of wild-type SpeA. These results illustrated that the yield of agmatine could be increased by releasing feedback inhibition of SpeA. In addition, the neutrality of fermentation broth by the production of agmatine relative to l-arginine may also be a reason for the high agmatine yield. The expression of SpeA mutant in the AGM3 strain was optimized by adding different concentrations of the inducer isopropyl β-d-1-thiogalactopyranoside (IPTG) at 24 h. And then, resulting in the production of 18.9 g/L of agmatine after fermentation of 96 h when 0.7 mM was added (Fig. 5c).

### Enhanced production of agmatine using fed-batch strategy

**Fig. 6:**
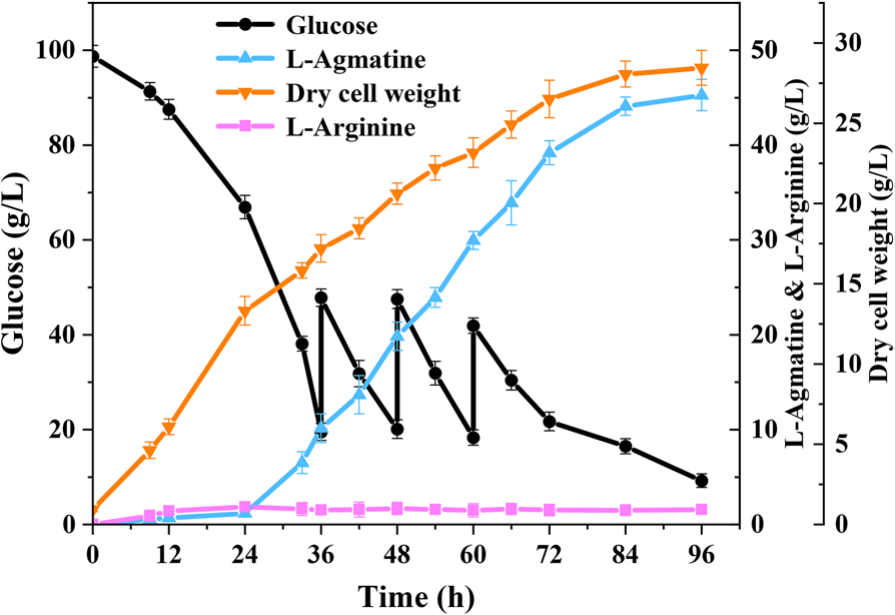
Time profile of agmatine production by recombinant *C. crenatum* AGM3 in fed-batch fermentation. All determinations were performed in triplicate

Finally, to further test the performance of engineered strain *C. crenatum* AGM3 for large-scale production of agmatine, fed-batch fermentations were carried out in a 5-L bioreactor with industrially relevant complex medium using glucose as carbon sources. The time profiles of fed-batch fermentation of the *C. crenatum* AGM3 were shown in Fig. 6. In the first stage (0–24 h), biomass was accumulated rapidly and achieved the DCW of 13.3 g/L, while glucose consumption was slow. The production of agmatine in the first stage may be due to the leaky expression of inducible promoter *tac*. With the induction of 0.7 mM IPTG at 24 h, a high accumulation of agmatine was observed with a high production rate up to 0.79 g/L/h during the phase of fermentation (24–72 h). Contrarily, the growth of the strain AGM3 became slower than the time before 24 h due to the addition of IPTG. At the end of fermentation, the *C. crenatum* AGM3 strain was able to produce 45.26 g/L of agmatine with the yield of 0.31 g/g glucose and achieved the productivity of 0.47 g/L/h. Different from shaking flask cultivations of the engineered strains AGM3, a small amount of extracellular intermediate metabolites l-arginine was produced in the culture medium before 24 h and maintained at a low-level titer of around 1.6 g/L during fed-batch fermentation. The maximum biomass was reached after 96 h fermentation and achieved the highest DCW of 28.44 g/L. Those are the highest value for agmatine production from *C. crenatum*, demonstrating that the engineered strain *C. crenatum* AGM3 is a competitive platform strain for agmatine production.

## Conclusions

In this study, *C. crenatum* was engineered for the first time to produce agmatine. The agmatine synthesis pathway was reconstructed and key catalytic steps were enhanced by screening efficient enzyme variants with high catalytic activity and agmatine tolerance. Compared with wild-type SpeA, the half-inhibitory concentration and specific enzyme activity of I534D were increased by 48.1% and 35.7%, respectively. The agmatine production of whole-cell bioconversion with AGM3 was 62.84 g/L in 12 h with a conversion rate of 83.44% and the yield was increased by 19.3% compared with AGM2. The final AGM3 strain can produce 45.26 g/L of agmatine with a yield of 0.31 g/g glucose in fed-batch fermentation. This result represented the highest yield of agmatine synthesized by *C. crenatum*. The present study also represents the first report on the efficient production of agmatine from low-value raw materials using *C. crenatum*, providing an avenue for the efficient industrial fermentative production of agmatine.

## Materials and methods

### Bacterial strains, plasmids and materials

The bacterial strains and plasmids used in this study are listed in Additional file 1: Table S1. The arginine decarboxylase was encoded by the genes of *speA* (Gene ID: 947432) and *adiA* (Gene ID: 948638) from *Escherichia coli* K-12. The strain used for heterologous expression was *E. coli* BL21 (DE3) and *C. crenatum* SYPA was used as an agmatine producer. The shuttle vector pXMJ19 was used for gene expression and mutagenesis studies in *E. coli* BL21 (DE3) and *C. crenatum* SYPA. DNA polymerases (2 × Phanta Max Master Mix, 2 × Taq Max Master Mix) used for PCR were purchased from Vazyme Biotech Co. Ltd. (Nanjing, China). Agmatine was purchased from Aladdin (Shanghai, China). All other chemicals of high grade were obtained from commercial sources.

### Structural analysis and molecular docking

The three-dimensional structure model of SpeA was downloaded from the Protein Data Bank (PDB) and PDB ID: 3NZQ [33]. The agmatine and l-arginine were docked into the protein structure by Schrodinger. The prime stability score of each docking intermediate complex was calculated, and the best docking position was selected by docking analysis. The best mutations score of prime stability were listed in Additional file 1: Table S2. Mutants of SpeA were modeled by introducing the mutated residues into the wild-type model.

### Cloning and site-directed mutagenesis of arginine decarboxylase

The *speA* gene coding for arginine decarboxylase was amplified by PCR using the *E. coli* genomic DNA as a template. The linear fragments of the amplified products were inserted into the *Bam*H I and *Eco*R I sites of the pXMJ19 vector. Mutations were introduced by overlapping PCR using the primers listed in Additional file 1: Table S3. The recombinant plasmids were finally transformed into *E. coli* BL21 (DE3) for expression and the success of the desired mutation was confirmed by DNA sequencing. The recombinant plasmids were transformed into *C. crenatum* SPYA using the electroporation method described by Tauch et al. [34].

### Expression and purification of proteins

All recombinant proteins expressed in *E. coli* or *C. crenatum* SPYA were labeled with a His-Tag at C-terminally. In order to express arginine decarboxylase, recombinant *E. coli* were cultured at 37 °C in LB medium until OD_600_ reached about 0.6. Then, isopropyl β-d-1-thiogalactopyranoside (IPTG) was added at a final concentration of 0.5 mM, and the strains were cultured at 16 °C for 12 h to induce the expression of the protein. The *C. crenatum* cells were cultured and induced in LBG medium for 18 h at 30 °C. After cultivation, the cells were collected by centrifugation at 8000×*g* and 4 °C for 5 min, washed twice and suspended in Tris-HCl buffer (pH 8.0). The cell suspension was disrupted on ice by sonication and the cell crude extract was obtained by centrifugation at 10,000×*g* and 4 °C for 20 min. After that, the proteins were purified using the HisTrap^TM^ HP affinity column (5 mL) and the AKTA protein purifier (GE Healthcare, Sweden). The target proteins were eluted by buffer M_0_ (20 mM Tris-HCl, 500 mM NaCl, pH 7.4) and M_700_ (20 mM Tris-HCl, 700 mM imidazole, 500 mM NaCl, pH 7.4) with a gradient ratio. The purified enzymes were analyzed by SDS-PAGE.

### Determining specific enzyme activity, biochemical properties and feedback inhibition of ADC

The enzyme activity of ADC was determined according to the amount of agmatine produced at 37 °C for 10 min [35]. The reaction mixture contained 50 mM Tris-HCl buffer (pH 8.0), 50 mM l-arginine, 2.5 mM MgSO_4_, 0.6 mM pyridoxal phosphate (PLP) and an appropriate amount of purified enzyme. The reaction was stopped by the addition of 500 μL 40% trichloroacetic acid. One unit of ADC activity is defined as the amount of enzyme that generated 1 µmol agmatine per minute. The l-arginine and agmatine concentrations were measured by HPLC. With bovine serum albumin (BSA) as the standard, the purified protein concentration was determined by Bradford [36].

The optimal temperature of ADC was determined by changing the temperature range from 30 °C to 70 °C. The optimum pH of ADC was measured by changing different pH values from 5.0 to 10.0. Thermostability was measured by incubating purified enzyme in the temperature range of 30 °C to 70 °C for 12 h at pH 8.0, then the residual enzyme activity was determined. In addition, the pH stability was determined by culturing purified enzyme in different pH buffers from 5.0 to 10.0 for 12 h. The activity of the enzyme without incubation was defined as 100%. The influence of metal ions on enzyme activity was measured using 1 mM Mg^2+^, Zn^2+^, Mn^2+^, Ca^2+^, Fe^2+^, Cu^2+^, Na^+^, Ni^2+^, Co^2+^, Li^+^, Fe^3+^, La^3+^, and K^+^ separately. The method of measuring enzyme activity was consistent with that described above. The activity of the enzyme without metal ions was defined as 100%.

The method of feedback inhibition determination was similar to that of SpeA activity determination, the only difference was that we added different concentrations of agmatine to the enzymatic reaction mixture. The inhibition curve was constructed by changing the concentration of agmatine in the reaction mixture. The activity of SpeA without agmatine was defined as 100%.

### Whole‑cell bioconversion and production of agmatine

A 50-mL whole-cell conversion was performed in a 250 mL airtight container. For the production of agmatine by whole-cell bioconversion, 50 g/L l-arginine was dissolved in 50 mM Tris-HCl buffer, including MgSO_4_, pyridoxal phosphate (PLP), and an appropriate amount of recombinant *C. crenatum* cells. Whole *C. crenatum* cells were added to this reaction solution with different final OD_600_ values (30, 35, 40, 45 and 50). The pH value of the reaction system was adjusted from 6.0 to 9.0. The different concentrations of PLP were added (0, 1, 3, 5, 7, 9 mM) and Mg^2+^ was added in concentrations of 1, 2, 3, 4 and 5 mM. After adding recombinant *C. crenatum* cells, the reaction solution was incubated at different temperatures (30 °C, 40 °C, 50 and 60 °C) for 12 h. After the reaction, the solution was centrifuged, and the product was analyzed by HPLC. The conversion rate is the molar concentration of the product to the molar concentration of the substrate.

### Fed-batch fermentation and medium

*Corynebacterium crenatum* SYPA as a chassis strain for agmatine production and LBG medium was used to culture *C. crenatum* and corresponding recombinant strains at 30 °C and 180 rpm. A single colony was inoculated into 20 mL of primary seed medium and cultured at 30 °C for 24 h in a 250 mL shake flask. The primary seed culture was inoculated to a 200 mL secondary seed medium and cultured at 30 °C. If necessary, 10 µg/mL chloramphenicol was added to the seed medium. After 12 h cultivation, 200 mL of the secondary seed culture was inoculated to a 5-L fermenter containing 2 L of the fermentation medium. The fermentation medium contained the following: 100 g/L glucose, 50 g/L corn steep liquor, 20 g/L yeast extract, 2 g/L KH_2_PO_4_, 0.5 g/L MgSO_4_·7H_2_O, 1 g/L KCl, 0.02 g/L FeSO_4_·7H_2_O, 0.02 g/L MnSO_4_, and 30 g/L (NH_4_)_2_SO_4_. The temperature and the airflow rate were maintained at 30 °C and 3 L/min, respectively. The pH was maintained at 7.0 by automatically adding 50% NH_3_·H_2_O solution. The feeding solution comprises the following components: 600 g/L glucose, 60 g/L corn steep liquor, 12 g/L KH_2_PO_4_, 3 g/L MgSO_4_·7H_2_O, 100 g/L (NH_4_)_2_SO_4_, 10 g/L yeast extract.

### Assays of cell concentration, glucose and agmatine

The absorbance at 562 nm wavelength (OD_562_, 1 OD_562_ = 0.375 g/L dry cell weight, DCW) was measured using Ultraviolet spectrophotometer (UNICOTM-UV2000, UNICO, Shanghai, China) for quantification of cell growth. The concentration of glucose in the fermentation supernatant were detected with SBA-40 C biosensor (developed by the Biology Institute of the Shandong Academy of Sciences, Jinan, China).

The concentration of agmatine and l-arginine was analyzed using the HPLC system (Agilent LC1260) equipped with an Agilent C18 (5 μm, 250 × 4.6 mm) and a UV detector. The chromatographic conditions were as follows: rate flow of 1 mL/min, temperature of 40 ℃ and wavelength of 338 nm. The agmatine and l-arginine were measured by OPA FMOC precolumn derivatization. The mobile phases required for agmatine and l-arginine detection were phase A and phase D. Phase A contained 8 g sodium acetate and 225 µL triethylamine per liter of aqueous solution, pH was adjusted to 7.2 with acetic acid, 5 mL tetrahydrofuran were added to the mixture. Phase D contained 6 g sodium acetate in 200 mL water, acetic acid was added to the solution and pH was controlled to 7.2, then 400 mL methanol, 400 mL acetonitrile were added.

## Supplementary Information

**Additional file 1: Table S1.** Strains and plasmids used in this study. **Table S2.** The score of prime stability after the mutation. **Table S3.** Primers used in this study.

## Data Availability

All data involved in this study are included in this published article [and its additional files].

## Abbreviations

ADC: Arginine decarboxylase
PLP: Pyridoxal phosphate
IPTG: Isopropyl-β-d-thiogalactopyranoside
HPLC: High-performance liquid chromatography
SDS-PAGE: Sodium dodecyl sulfate polyacrylamide gel electrophoresis
