# Supplementary material for: High-level production of the agmatine in engineered Corynebacterium crenatum with the inhibition-releasing arginine decarboxylase
Source: Microb Cell Fact. 2022 Jan 31;21:16. doi: 10.1186/s12934-022-01742-3 (PMC8805389; doi:10.1186/s12934-022-01742-3)
Supplement: Supplementary file 1 — Additional file 1: Table S1. Strains and plasmids used in this study. Table S2. The score of prime stability after the mutation. Table S3. Primers used in this study. [file 12934_2022_1742_MOESM1_ESM.docx]

**Supporting Information**

**High-level Production of the Agmatine in Engineered** ***Corynebacterium crenatum* with the** **inhibition-****releasing Arginine decarboxylase**

Fengyu Yang^a^, Jiayu Xu^a^, Yichun Zhu^a^, Yi Wang^a^, Meijuan Xu^a^*, Zhiming Rao^a^*

^a^Key Laboratory of Industrial Biotechnology of the Ministry of Education, School of Biotechnology, Jiangnan University, Wuxi, Jiangsu 214122, China

*Corresponding authors:

Meijuan Xu, E-mail: [xumeijuan@jiangnan.edu.cn](mailto:xumeijuan@jiangnan.edu.cn), Tel: 86-510-85916881

ZhiMing Rao, E-mail: [raozhm@jiangnan.edu.cn](mailto:raozhm@jiangnan.edu.cn), Tel: 86-510-85916881

Email address:

534842301@qq.com (F. Y. Yang); 1753175712@qq.com (J. Y. Xu)

1022190205@stu.jiangnan.edu.cn (Y. C. Zhu); onsewan94@gmail.com (Y. Wang)

xumeijuan@jiangnan.edu.cn (M. J. Xu); raozhm@jiangnan.edu.cn (Z. M. Rao)

**Table S1** **Strains and plasmids used in this study.**

| **Strains or plasmid** | **Description** | **Source or reference** | |
| --- | --- | --- | --- |
| **Strains** | | | |
| *E. coli* | | | |
| BL21 (DE3) | Host for recombinant protein production | | Laboratory stock |
| BL21/pXMJ19 | BL21 harboring pXMJ19 | | This work |
| BL21/pXMJ19-*speA* | BL21 harboring pXMJ19-*speA* | | This work |
| BL21/pXMJ19-*adiA* | BL21 harboring pXMJ19-*adiA* | | This work |
| BL21/pXMJ19-*speA*_D531P_ | BL21 harboring pXMJ19-*speA*_D531P_ | | This work |
| BL21/pXMJ19-*speA*_D531E_ | BL21 harboring pXMJ19-*speA*_D531E_ | | This work |
| BL21/pXMJ19-*speA*_A533P_ | BL21 harboring pXMJ19-*speA*_A533P_ | | This work |
| BL21/pXMJ19-*speA*_D535W_ | BL21 harboring pXMJ19-*speA*_D535W_ | | This work |
| BL21/pXMJ19-*speA*_D535E_ | BL21 harboring pXMJ19-*speA*_D535E_ | | This work |
| BL21/pXMJ19-*speA*_D534K_ | BL21 harboring pXMJ19-*speA*_D534K_ | | This work |
| BL21/pXMJ19-*speA*_A534T_ | BL21 harboring pXMJ19-*speA*_A534T_ | | This work |
| BL21/pXMJ19-*speA*_D534C_ | BL21 harboring pXMJ19-*speA*_D534C_ | | This work |
| BL21/pXMJ19-*speA*_D534N_ | BL21 harboring pXMJ19-*speA*_D534N_ | | This work |
| BL21/pXMJ19-*speA*_A534A_ | BL21 harboring pXMJ19-*speA*_A534A_ | | This work |
| BL21/pXMJ19-*speA*_D534S_ | BL21 harboring pXMJ19-*speA*_D534S_ | | This work |
| BL21/pXMJ19-*speA*_D534G_ | BL21 harboring pXMJ19-*speA*_D534G_ | | This work |
| BL21/pXMJ19-*speA*_A534Y_ | BL21 harboring pXMJ19-*speA*_A534Y_ | | This work |
| BL21/pXMJ19-*speA*_D534E_ | BL21 harboring pXMJ19-*speA*_D534E_ | | This work |
| BL21/pXMJ19-*speA*_D534P_ | BL21 harboring pXMJ19-*speA*_D534P_ | | This work |
| BL21/pXMJ19-*speA*_A534D_ | BL21 harboring pXMJ19-*speA*_A53D_ | | This work |
| *C. crenatum* | | | |
| *C. crenatum* SYPA | L-arginine-producing strain (CCTCC AB 2021051) | | Laboratory stock |
| AGM0 | SYPA harboring pXMJ19 | | This work |
| AGM1 | SYPA harboring pXMJ19-*adiA* | | This work |
| AGM2 | SYPA harboring pXMJ19-*speA* | | This work |
| AGM3 | SYPA harboring pXMJ19-*speA*_I534D_ | | This work |
| **Plasmid** | | | |
| pXMJ19 | Chl^r^; shuttle vector between *E. coli* and *C. crenatum* | | Laboratory stock |
| pXMJ19-*speA* | derived from pXMJ19, for expression of *speA* | | This work |
| pXMJ19-*adiA* | derived from pXMJ19, for expression of *adiA* | | This work |
| pXMJ19-*speA*_D513P_ | derived from pXMJ19, for expression of *speA*_D513P_ | | This work |
| pXMJ19-*speA*_D531E_ | derived from pXMJ19, for expression of *speA*_D531E_ | | This work |
| pXMJ19-*speA*_A533P_ | derived from pXMJ19, for expression of *speA*_A533P_ | | This work |
| pXMJ19-*speA*_D535W_ | derived from pXMJ19, for expression of *speA*_D535W_ | | This work |
| pXMJ19-*speA*_D535E_ | derived from pXMJ19, for expression of *speA*_D535E_ | | This work |
| pXMJ19-*speA*_I534K_ | derived from pXMJ19, for expression of *speA*_I534K_ | | This work |
| pXMJ19-*speA*_I534T_ | derived from pXMJ19, for expression of *speA*_I534T_ | | This work |
| pXMJ19-*speA*_I534C_ | derived from pXMJ19, for expression of *speA*_I534C_ | | This work |
| pXMJ19-*speA*_I534N_ | derived from pXMJ19, for expression of *speA*_I534N_ | | This work |
| pXMJ19-*speA*_I534A_ | derived from pXMJ19, for expression of *speA*_I534A_ | | This work |
| pXMJ19-*speA*_I534S_ | derived from pXMJ19, for expression of *speA*_I534S_ | | This work |
| pXMJ19-*speA*_I534G_ | derived from pXMJ19, for expression of *speA*_I534G_ | | This work |
| pXMJ19-*speA*_I534Y_ | derived from pXMJ19, for expression of *speA*_I534Y_ | | This work |
| pXMJ19-*speA*_I534E_ | derived from pXMJ19, for expression of *speA*_I534E_ | | This work |
| pXMJ19-*speA*_I534P_ | derived from pXMJ19, for expression of *speA*_I534P_ | | This work |
| pXMJ19-*speA*_I534D_ | derived from pXMJ19, for expression of *speA*_I534D_ | | This work |

**Table S2** **The score of prime stability after the mutation.**

|  | **Score** | **Effect** |
| --- | --- | --- |
| WT | 5927.93 | Stabilizing |
| D531P | 5923.57 | Stabilizing |
| D531E | 5899.49 | Stabilizing |
| A533P | 5926.448 | Stabilizing |
| D535W | 5901.79 | Stabilizing |
| D535E | 5901.07 | Stabilizing |
| I534K | 5930.21 | Stabilizing |
| I534T | 5931.68 | Stabilizing |
| I534C | 5932.77 | Stabilizing |
| I534N | 5933.01 | Stabilizing |
| I534A | 5934.84 | Stabilizing |
| I534S | 5938.09 | Stabilizing |
| I534G | 5938.74 | Stabilizing |
| I534Y | 5938.76 | Stabilizing |
| I534E | 5942.08 | Stabilizing |
| I534P | 5953.87 | Stabilizing |
| I534D | 5957.32 | Stabilizing |

**Table S3** **Primers used in this study.**

| **Primer** | **Sequence (5’-3’)** | **Description** |
| --- | --- | --- |
| *speA*-F | gcaggtcgactctagaggatccaaaggaggaaaatcatgtctgacgacatgtctatggg | Amplification of *speA* for construction of pXMJ19-*speA* |
| *speA*-R | gccaaaacagccaagctgaattcttagtggtggtggtggtggtgctcatcttcaagataagtataaccgtacaaacc |  |
| *adiA*-F | ggtcgactctagaggatccaaaggaggacaaccatgaaagtattaattgttgaaagcgagtttc | Amplification of *adiA* for construction of pXMJ19-*adiA* |
| *adiA*-R | gccaagctgaattcgagctcttagtggtggtggtggtggtgcgctttcacgcacataacg |  |
| *speA*_D531P_-A | gcaggtcgactctagaggatccaaaggaggaaaatcatgtctgacgacatgtctatggg | Amplification of *speA* with D531P mutant for construction of pXMJ19-*speA*_D531P_ |
| *speA*_D531P_-B | caatatagtggtcgatagcacc**cgg**agagtcacaggtaatatc |  |
| *speA*_D531P_-C | gatattacctgtgactct**ccg**ggtgctatcgaccactatattg |  |
| *speA*_D531P_-D | gccaaaacagccaagctgaattcttagtggtggtggtggtggtgctcatcttcaagataagtataaccgtacaaacc |  |
| *speA*_D531E_-A | gcaggtcgactctagaggatccaaaggaggaaaatcatgtctgacgacatgtctatggg | Amplification of *speA* with D531E mutant for construction of pXMJ19-*speA*_D531E_ |
| *speA*_D531E_-B | caatatagtggtcgatagcacc**ctc**agagtcacaggtaatatc |  |
| *speA*_D531E_-C | gatattacctgtgactct**gag**ggtgctatcgaccactatattg |  |
| *speA*_D531E_-D | gccaaaacagccaagctgaattcttagtggtggtggtggtggtgctcatcttcaagataagtataaccgtacaaacc |  |
| *speA*_A533P_-A | gcaggtcgactctagaggatccaaaggaggaaaatcatgtctgacgacatgtctatggg | Amplification of *speA* with A533P mutant for construction of pXMJ19-*speA*_A533P_ |
| *speA*_A533P_-B | ccatcaatatagtggtcgat**cgg**accgtcagagtcacagg |  |
| *speA*_A533P_-C | cctgtgactctgacggt**ccg**atcgaccactatattgatgg |  |
| *speA*_A533P_-D | gccaaaacagccaagctgaattcttagtggtggtggtggtggtgctcatcttcaagataagtataaccgtacaaacc |  |
| *speA*_D535W_-A | gcaggtcgactctagaggatccaaaggaggaaaatcatgtctgacgacatgtctatggg | Amplification of *speA* with D535W mutant for construction of pXMJ19-*speA*_D535W_ |
| *speA*_D535W_-B | caccatcaatatagtg**cca**gatagcaccgtcagagtcac |  |
| *speA*_D535W_-C | gtgactctgacggtgctatc**tgg**cactatattgatggtg |  |
| *speA*_D535W_-D | gccaaaacagccaagctgaattcttagtggtggtggtggtggtgctcatcttcaagataagtataaccgtacaaacc |  |
| *speA*_D535E_-A | gcaggtcgactctagaggatccaaaggaggaaaatcatgtctgacgacatgtctatggg | Amplification of *speA* with D535E mutant for construction of pXMJ19-*speA*_D535E_ |
| *speA*_D535E_-B | caccatcaatatagtg**ctc**gatagcaccgtcagagtcac |  |
| *speA*_D535E_-C | gtgactctgacggtgctatc**gag**cactatattgatggtg |  |
| *speA*_D535E_-D | gccaaaacagccaagctgaattcttagtggtggtggtggtggtgctcatcttcaagataagtataaccgtacaaacc |  |
| *speA*_I534K_-A | gcaggtcgactctagaggatccaaaggaggaaaatcatgtctgacgacatgtctatggg | Amplification of *speA* with I534K mutant for construction of pXMJ19-*speA*_I534K_ |
| *speA*_I534K_-B | caccatcaatatagtggtc**ttt**agcaccgtcagagtcac |  |
| *speA*_I534K_-C | gtgactctgacggtgct**aaa**gaccactatattgatggtg |  |
| *speA*_I534K_-D | gccaaaacagccaagctgaattcttagtggtggtggtggtggtgctcatcttcaagataagtataaccgtacaaacc |  |
| *speA*_I534T_-A | gcaggtcgactctagaggatccaaaggaggaaaatcatgtctgacgacatgtctatggg | Amplification of *speA* with I534T mutant for construction of pXMJ19-*speA*_I534T_ |
| *speA*_I534T_-B | caccatcaatatagtggtc**agt**agcaccgtcagagtcac |  |
| *speA*_I534T_-C | gtgactctgacggtgct**act**gaccactatattgatggtg |  |
| *speA*_I534T_-D | gccaaaacagccaagctgaattcttagtggtggtggtggtggtgctcatcttcaagataagtataaccgtacaaacc |  |
| *speA*_I534C_-A | gcaggtcgactctagaggatccaaaggaggaaaatcatgtctgacgacatgtctatggg | Amplification of *speA* with I534C mutant for construction of pXMJ19-*speA*_I534C_ |
| *speA*_I534C_-B | caccatcaatatagtggtc**gca**agcaccgtcagagtcac |  |
| *speA*_I534C_-C | gtgactctgacggtgct**tgc**gaccactatattgatggtg |  |
| *speA*_I534C_-D | gccaaaacagccaagctgaattcttagtggtggtggtggtggtgctcatcttcaagataagtataaccgtacaaacc |  |
| *speA*_I534N_-A | gcaggtcgactctagaggatccaaaggaggaaaatcatgtctgacgacatgtctatggg | Amplification of *speA* with I534N mutant for construction of pXMJ19-*speA*_I534N_ |
| *speA*_I534N_-B | gtgactctgacggtgct**aac**gaccactatattgatggtg |  |
| *speA*_I534N_-C | caccatcaatatagtggtc**gtt**agcaccgtcagagtcac |  |
| *speA*_I534N_-D | gccaaaacagccaagctgaattcttagtggtggtggtggtggtgctcatcttcaagataagtataaccgtacaaacc |  |
| *speA*_I534A_-A | gcaggtcgactctagaggatccaaaggaggaaaatcatgtctgacgacatgtctatggg | Amplification of *speA* with I534A mutant for construction of pXMJ19-*speA*_I534A_ |
| *speA*_I534A_-B | caccatcaatatagtggtc**agc**agcaccgtcagagtcac |  |
| *speA*_I534A_-C | gtgactctgacggtgct**gct**gaccactatattgatggtg |  |
| *speA*_I534A_-D | gccaaaacagccaagctgaattcttagtggtggtggtggtggtgctcatcttcaagataagtataaccgtacaaacc |  |
| *speA*_I534S_-A | gcaggtcgactctagaggatccaaaggaggaaaatcatgtctgacgacatgtctatggg | Amplification of *speA* with I534S mutant for construction of pXMJ19-*speA*_I534S_ |
| *speA*_I534S_-B | caccatcaatatagtggtc**cga**agcaccgtcagagtcac |  |
| *speA*_I534S_-C | gtgactctgacggtgct**tcg**gaccactatattgatggtg |  |
| *speA*_I534S_-D | gccaaaacagccaagctgaattcttagtggtggtggtggtggtgctcatcttcaagataagtataaccgtacaaacc |  |
| *speA*_I534G_-A | gcaggtcgactctagaggatccaaaggaggaaaatcatgtctgacgacatgtctatggg | Amplification of *speA* with I534G mutant for construction of pXMJ19-*speA*_I534G_ |
| *speA*_I534G_-B | caccatcaatatagtggtc**acc**agcaccgtcagagtcac |  |
| *speA*_I534G_-C | gtgactctgacggtgct**ggt**gaccactatattgatggtg |  |
| *speA*_I534G_-D | gccaaaacagccaagctgaattcttagtggtggtggtggtggtgctcatcttcaagataagtataaccgtacaaacc |  |
| *speA*_I534Y_-A | gcaggtcgactctagaggatccaaaggaggaaaatcatgtctgacgacatgtctatggg | Amplification of *speA* with I534Y mutant for construction of pXMJ19-*speA*_I534Y_ |
| *speA*_I534Y_-B | caccatcaatatagtggtc**gta**agcaccgtcagagtcac |  |
| *speA*_I534Y_-C | gtgactctgacggtgct**tac**gaccactatattgatggtg |  |
| *speA*_I534Y_-D | gccaaaacagccaagctgaattcttagtggtggtggtggtggtgctcatcttcaagataagtataaccgtacaaacc |  |
| *speA*_I534E_-A | gcaggtcgactctagaggatccaaaggaggaaaatcatgtctgacgacatgtctatggg | Amplification of *speA* with I534E mutant for construction of pXMJ19-*speA*_I534E_ |
| *speA*_I534E_-B | caccatcaatatagtggtc**ctc**agcaccgtcagagtcac |  |
| *speA*_I534E_-C | gtgactctgacggtgct**gag**gaccactatattgatggtg |  |
| *speA*_I534E_-D | gccaaaacagccaagctgaattcttagtggtggtggtggtggtgctcatcttcaagataagtataaccgtacaaacc |  |
| *speA*_I534P_-A | gcaggtcgactctagaggatccaaaggaggaaaatcatgtctgacgacatgtctatggg | Amplification of *speA* with I534P mutant for construction of pXMJ19-*speA*_I534P_ |
| *speA*_I534P_-B | caccatcaatatagtggtc**agg**agcaccgtcagagtcac |  |
| *speA*_I534P_-C | gtgactctgacggtgct**cct**gaccactatattgatggtg |  |
| *speA*_I534P_-D | gccaaaacagccaagctgaattcttagtggtggtggtggtggtgctcatcttcaagataagtataaccgtacaaacc |  |
| *speA*_I534D_-A | gcaggtcgactctagaggatccaaaggaggaaaatcatgtctgacgacatgtctatggg | Amplification of *speA* with I534D mutant for construction of pXMJ19-*speA*_I534D_ |
| *speA*_I534D_-B | caccatcaatatagtggtc**atc**agcaccgtcagagtcac |  |
| *speA*_I534D_-C | gtgactctgacggtgct**gat**gaccactatattgatggtg |  |
| *speA*_I534D_-D | gccaaaacagccaagctgaattcttagtggtggtggtggtggtgctcatcttcaagataagtataaccgtacaaacc |  |
